# Regulation of Ferroptosis by Non-Coding RNAs in Head and Neck Cancers

**DOI:** 10.3390/ijms23063142

**Published:** 2022-03-15

**Authors:** Pei-Ling Hsieh, Shih-Chi Chao, Pei-Ming Chu, Cheng-Chia Yu

**Affiliations:** 1 Department of Anatomy, School of Medicine, China Medical University, Taichung 404333, Taiwan; plhsieh@mail.cmu.edu.tw (P.-L.H.); pmchu@mail.cmu.edu.tw (P.-M.C.); 2 Institute of Oral Sciences, Chung Shan Medical University, Taichung 40201, Taiwan; h5l4g4fu6123@gmail.com; 3 Department of Medical Research and Education, Lo-Hsu Medical Foundation, Lotung Poh-Ai Hospital, Yilan 265, Taiwan; 4 School of Dentistry, Chung Shan Medical University, Taichung 40201, Taiwan; 5 Department of Dentistry, Chung Shan Medical University Hospital, Taichung 40201, Taiwan

**Keywords:** ferroptosis, non-coding RNAs, head and neck cancers

## Abstract

Ferroptosis is a newly identified mode of programmed cell death characterized by iron-associated accumulation of lipid peroxides. Emerging research on ferroptosis has suggested its implication in tumorigenesis and stemness of cancer. On the other hand, non-coding RNAs have been shown to play a pivotal role in the modulation of various genes that affect the progression of cancer cells and ferroptosis. In this review, we summarize recent advances in the theoretical modeling of ferroptosis and its relationship between non-coding RNAs and head and neck cancers. Aside from the significance of ferroptosis-related non-coding RNAs in prognostic relevance, we also review how these non-coding RNAs participate in the regulation of iron, lipid metabolism, and reactive oxygen species accumulation. We aim to provide a thorough grounding in the function of ferroptosis-related non-coding RNAs based on current knowledge in an effort to develop effective therapeutic strategies for head and neck cancers.

## 1. Ferroptosis

In 2012, Dixon et al. proposed the concept of ferroptosis, an iron-dependent, oxidative form of non-apoptotic cell death that is triggered by a RAS-selective lethal small molecule erastin and can be prevented by iron chelators [1]. There are two major categories of cell death, accidental cell death (ACD) and regulated cell death (RCD), and ferroptosis is defined as a type of RCD [2]. Unlike other types of RCD, ferroptosis does not express the following morphological features such as cytoplasmic shrinkage, chromatin condensation, nuclear fragmentation, formation of cytoplasmic vacuolization, swelling of the cytoplasm and organelles, and rupture of the cell membrane. Ferroptosis can be initiated by the inhibition of system xc−, the cystine-glutamate antiporter, which limits the availability of glutathione (GSH) [3]. As a cofactor for glutathione peroxidase 4 (GPX4), depletion of GSH results in the decreased activity of this lipid-repairing enzyme and the subsequent accumulation of lipid-based reactive oxygen species (ROS), particularly lipid peroxides as GPX4 converts GSH into oxidized glutathione and reduces the cytotoxic lipid hydroperoxide peroxides (L-OOH) to the non-toxic lipid alcohols (L-OH). Apart from GPX4, another anti-ferroptosis pathway has recently been identified. It has been shown that ferroptosis suppressor protein 1 (FSP1, previously known as apoptosis-inducing factor mitochondria-associated 2 (AIFM2)), inhibits ferroptosis and lipid peroxidation by acting as an NADH-dependent coenzyme Q_10_ (CoQ_10_) oxidoreductase [4,5]. On the other hand, iron metabolism plays an important role in ferroptosis. It has been demonstrated that administration of iron chelators blocks ferroptosis and supplement of exogenous iron enhances the sensitivity of cells to ferroptosis inducers [1]. Although the detailed mechanisms underlying the role of iron in ferroptosis remain obscure, it has been considered that the excess iron may donate electrons to O_2_ and H_2_O_2_ to generate superoxide anions and the hydroxyl radical (OH) in the Fenton reaction, leading to the oxidization of proteins, lipids, and nucleic acids.

### 1.1. Iron Metabolism

Iron has a fundamental role in a wide variety of biochemical reactions such as electron transport, oxygen transport, deoxyribonucleotide synthesis, and various reactions involving three major iron-containing proteins, heme-containing proteins, iron–sulfur (Fe–S) cluster proteins, and other iron-containing enzymes. Mitochondria are the site responsible for the synthesis of heme and Fe–S clusters, and the iron entry into mitochondria is mediated by various transporters including mitoferrin1 and mitoferrin2 in vertebrates. Mitoferrin-1 (Mfrn1; encoded by the gene solute carrier family 25 member 37, Slc25a37), is localized in the mitochondrial inner membrane and highly expressed in developing erythroid cells, whereas Mitoferrin2 (Mfrn2; Slc25a28) seems to be ubiquitously expressed. Under physiological conditions, ferric iron (Fe^3+^) is carried by the iron-binding protein transferrin (Tf), which is selective for Fe^3+^ and can bind two Fe^3+^ per Tf. Once the Tf–Fe^3+^ complex binds to its cognate receptor, transferrin receptor-1 (TfR1), the complex of Tf–Fe^3+^TfR1 is then internalized into endosomes. Afterward, the dissociation of iron from Tf occurs when Fe^3+^ is reduced to ferrous iron (Fe^2+^), which can be transported to the cytosol via the divalent metal transporter l (DMT1), sequestered by ferritin, and exported into the extracellular fluid by ferroportin [6]. Aside from Tf, lactotransferrin (also known as lactoferrin; LTF) is another 80-kD extracellular iron-binding glycoprotein found in numerous mucosal secretions such as tears, saliva, vaginal fluids, and notably in milk and colostrum [7], and aids in the increased intracellular iron during chronic inflammation and tissue injury.

### 1.2. Lipid Metabolism

Lipid peroxidation is associated with the oxidation of polyunsaturated fatty acid (PUFA), and the hydroxyl radical and the hydroperoxyl radical (∙OOH) are strong initiators of the chain oxidation of PUFA in a non-enzymatic manner. Furthermore, lipids can be oxidized by numerous enzymes such as lipoxygenases (LOXs) [8] and cytochrome P450 [9]. It has been known that the secondary toxic derivatives of lipid peroxidation such as malondialdehyde (MDA) and 4-hydroxynonenal (4-HNE) can modulate multiple protein functions and signaling pathways that interfere with cell death [10]. Additionally, these reactive aldehydes have been identified in various modes of ferroptosis [9,11]. It has been suggested that lipid peroxidation may disturb membrane lipid composition, leading to alteration of ion gradients, membrane fluidity, and membrane permeability [12]. In addition, incorporation of long PUFAs into cellular membranes by acyl-CoA synthetase long-chain family member 4 (ACSL4) promotes ferroptosis execution [13]. Furthermore, it has been demonstrated that inhibition of ACSL4 downregulates the esterification of two main PUFAs, arachidonoyl (AA) and adrenoyl (AdA), into phosphatidylethanolamines (PE), and the suppression of iron-containing LOXs protect cells against the following ferroptotic signals after oxidation of AA [14]. Moreover, it has been shown that AMP-activated protein kinase (AMPK) mediates ferroptosis via AMPK-mediated phosphorylation of acetyl-CoA carboxylase (ACC) and PUFA biosynthesis [15]. Liver kinase B1 (LKB1) is an upstream kinase of AMPK activation, and the LKB1–AMPK axis has been demonstrated to negatively regulate ferroptosis by inhibitory phosphorylation of ACC1 and the following lipid hydroperoxidation [16].

### 1.3. Ferroptosis and Cancers

Multiple epidemiological and animal studies have suggested that excess iron is a major risk for carcinogenesis via the increased iron-catalyzed free radical-mediated oxidative stress [17,18]. Of relevance for cancer therapy, various studies have demonstrated that treatments that induced ferroptosis in resistant head and neck cells potentiate the treatment effects by GSH downregulation and ROS accumulation [19,20,21]. Cancer stem cells (CSCs), a small subpopulation of cells within the tumors, are considered to initiate tumor growth via the ability to self-renew and differentiate into the bulk of the tumor mass. Aside from driving tumor growth, CSCs are also implicated in drug resistance and metastasis. One recent study has shown that exposure to iron promotes the exhibition of CSC features in non-small cell lung carcinoma cells, and a higher level of hydroxyl radical correlates with the increased CSC phenotypes. Moreover, the stem cell/CSC marker, sex-determining region Y-box 9 protein (SOX9), is associated with the CSC enrichment mediated by iron [22]. It has been reported that there is an increased secretion of Tf, expression of TfR1, and iron uptake in CSCs of glioblastoma [23]. Additionally, their work demonstrated that silencing ferritin expression decreases CSCs growth in vitro and in vivo [23]. Another study showed that a synthetic derivative of salinomycin triggers the iron-mediated production of ROS in lysosomes, leading to a cell death pathway consistent with ferroptosis in breast CSCs [24].

Numerous markers have been uncovered to identify these CSCs in several types of cancers. For instance, CD44 (a cell surface adhesion receptor) [25], CD133 (a pentaspan membrane glycoprotein) [26], and aldehyde dehydrogenase 1 (ALDH1; enzymes that oxidize intracellular aldehydes and contribute to the oxidation of retinol to retinoic acid) [27] have been employed to recognize CSCs in head and neck cancers. It has been revealed that a CD44 variant (CD44v) interacts with and stabilizes xCT, a component of system xc−, which controls the GSH synthesis [28]. Ishimoto et al. showed that CSCs with a higher expression of CD44 demonstrated an increased capacity to synthesize GSH and suppress ROS. In addition, loss of xCT by ablation of CD44 retarded the tumor growth in a transgenic mouse model of gastric cancer [28]. Accordingly, treatments targeted to the CD44v–xCT axis may impair the ROS defense capacity of CSCs. Indeed, it has been shown that pharmacological and genetic inhibition of cystine/glutamate antiporter markedly sensitized resistant head and neck cells to cisplatin in vitro and in vivo [29]. Regarding CD133, it has been revealed to play a significant role in the uptake of Tf and iron metabolism in human colon cancer cells [30]. Moreover, ALDH1 has been proven to confer erlotinib (an epidermal growth factor receptor tyrosine-kinase inhibitor) resistance via upregulation of antioxidant enzyme, superoxidase dismutase (SOD)2, and GPX4, which downregulated the erlotinib-induced oxidative and carbonyl stress in lung adenocarcinomas [31].

## 2. Head and Neck Cancers and Non-Coding RNAs

### 2.1. Head and Neck Cancers

Head and neck cancers such as nasopharyngeal carcinoma (NPC) or oral squamous cell carcinoma (OSCC) often refer to cancers occurring from the squamous cells of mucosal surfaces in the following anatomical sites, the oral cavity, sinonasal cavity, pharynx, and larynx. These cancers are common malignancies worldwide and are often associated with areca nut [32]/tobacco [33] consumption, alcohol abuse [34], or infection with human papillomavirus (HPV) such as HPV-16 [35]. Furthermore, men are more likely to have head and neck cancers than women [36]. Head and neck cancers accounted for about 610,000 new cases and 306,000 deaths in 2020 according to GLOBOCAN 2020 [37]. A significant number of patients with advanced head and neck squamous cell carcinomas (HNSCC) presented with a higher risk of local recurrence, distant metastasis, and a poor prognosis (5-year overall survival is approximately 50%) [38]. One of the recent reports revealed that around 4% of patients with locoregionally controlled oral carcinoma developed distant metastasis, and the lung was the most common site [39]. The first-line standard-of-care therapy has been established by demonstrating that cetuximab added to chemotherapy consisting of fluorouracil plus a platinum (cisplatin or carboplatin) markedly increased overall survival, progression-free survival, and the overall response rate compared with chemotherapy alone [40,41]. Nevertheless, the need for weekly administration of cetuximab led to several unfavorable results such as infusion reactions and skin reactions, which greatly affected their quality of life. Accordingly, considerable efforts have been made to elucidate the pathophysiology of HNSCC and the emerging evidence indicates the key roles of non-coding RNAs in the development and progression of HNSCC.

### 2.2. Non-Coding RNAs

Non-coding RNAs are genes that produce functional RNA molecules instead of encoding proteins. Multiple categories have been discovered such as small non-coding RNAs (e.g., microRNAs; ~18–25 nucleotides in length), long non-coding RNAs (lncRNAs; >200 nucleotides in length), or circular RNA (circRNAs; a closed-loop structure microRNA sponge). It has been shown that microRNAs bind to the 3′ untranslated region (3′ UTR) of the target mRNAs, leading to translation inhibition or mRNA degradation [42]. LncRNAs have been shown to exert biological activities through a variety of mechanisms including serving as scaffolds, molecular signals, guides, or decoys [43]. Regarding circRNAs, it has been revealed to form covalently closed-loop structures with neither 5′–3′ polarities nor polyadenylated tails [44]. Both lncRNAs and circRNAs have been demonstrated to act as competing endogenous RNAs (ceRNAs) and regulate the distribution of miRNAs via miRNA response elements [45,46], which titrate miRNAs away from natural targets. Hence, lncRNAs and circRNAs are called the “sponge” of microRNAs by harboring multiple miRNA binding sites to repress the activity of one or multiple miRNAs. Similar to lncRNAs, circRNAs can directly bind to downstream proteins and affect their functions. Numerous reviews have summarized the potential contribution of these non-coding RNAs in the malignant progression of HNSCC via regulation of cell proliferation, metastasis potential, glucose metabolism, drug resistance, cancer stemness, and so on [47,48,49]. Likewise, accumulating data indicated that non-coding RNAs may also play a significant role in the regulation of ferroptosis in HNSCC, so we briefly discussed the research progress and aimed to explore and identify the possible molecular markers and therapeutic targets.

## 3. Ferroptosis-Associated Non-Coding RNAs in Head and Neck Cancers

**Figure 1:**
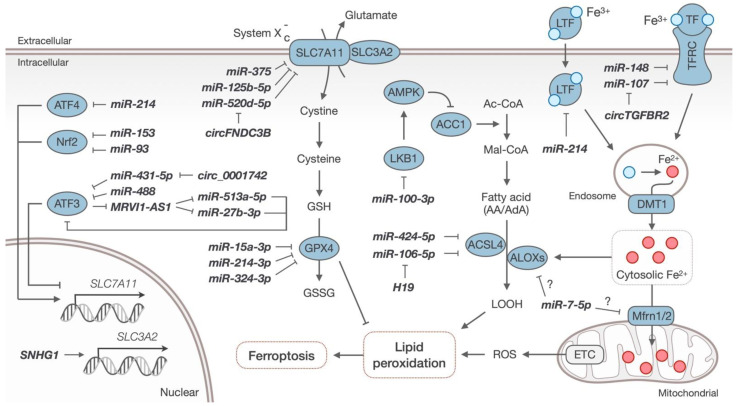
Roles and functions of the listed ncRNAs in the regulation of ferroptosis in head and neck cancers.

Over the past years, research has focused intensely on non-coding RNAs and ferroptosis. In this manuscript, we have summarized how these non-coding RNAs are implicated in various aspects of ferroptosis in head and neck cancers (Table 1 and Figure 1) including iron metabolism (Mfrn1/2, LOX, TfR1, and LTF), lipid metabolism (LKB1 and ACSL4), and ROS accumulation (SLC3A2, SLC7A11, ATF3, Nrf2, and GPX4).

### 3.1. Prognosis Roles of Ferroptosis-Related Non-Coding RNAs

Emerging evidence indicates that the ferroptosis-related lncRNAs play pivotal roles in the prognostic prediction of various types of cancers including HNSCC. A number of studies have characterized the differentially expressed ferroptosis-related genes using various databases to predict the potential targets. For instance, one recent report suggests that there are six autophagy- and ferroptosis-related-lncRNAs that may confer tumorigenesis in HNSCC including MIR4435-2HG, PCED1B-AS1, AL512274.1, LINC02541, AL354836.1, and MIR9-3HG [87]. Among the 926 ferroptosis-related lncRNAs in head and neck cancers, six lncRNAs (SLCO4A1-AS1, C1RL-AS1, PCED1B-AS1, HOXB-AS3, MIR9-3HG, and SFTA1P) have been suggested to possess the potential to predict prognostic risks and survival outcomes [88]. Another study showed that several lncRNAs that were associated with ferroptosis such as LINC01963, LINC01980, AATBC, ELF3-AS1, PSMA3-AS1, PCED1B-AS1, EP300-AS1, and PAX8-AS1 can serve as independent prognosis predictors of head and neck cancers [89].

Although these ferroptosis-related lncRNAs appear to involve in tumor progression, there is a lack of information to demonstrate whether they participate in tumorigenesis through the regulation of ferroptosis. Currently, only a couple of lncRNAs have been investigated. For example, it has been shown that MIR4435-2HG expression was markedly upregulated in HNSCC tissues and contributed to tumor progression via regulation of the miR-383-5p/RNA-binding motif protein 3 (RBM3) axis [90]. SLCO4A1-AS1 was overexpressed in laryngeal squamous cell carcinoma tissues and promoted cell proliferation by modulation of the miR-7855-5p/SETD7 axis and activation of the Wnt/β-catenin pathway [91]. In OSCC, HOXB-AS3 was upregulated in clinical tissues and exerted its oncogenic effect via direct interaction with insulin-like growth factor 2 mRNA-binding protein 2 (IGF2BP2) and thereby promoted the stability of c-myc [92]. Regarding ELF3-AS1, it has been shown to enhance the proliferation of OSCC cells by reprogramming glucose metabolism [93]. Another research demonstrated that PSMA3-AS1 functions as a contributing factor to facilitate malignant behaviors of OSCC cells via the miR-136-5p/fibronectin1 axis [94]. It is worthy of investigation to examine whether targeting these ferroptosis-related lncRNAs can achieve remission through ferroptosis regulation.

Virus infection is considered high risk for head and neck cancers. Patients who have heavy use of tobacco/alcohol or HPV infection or infected with herpes simplex virus (HSV) had lowered risk of head and neck cancer than those who were not infected [95]. Although the roles of HSV infection on head and neck cancer are still unknown, the underlying mechanism may be to modulate the ferroptosis, which leads head and neck cancer to be sensitized to radio- and chemotherapy [96]. HSV-1 infection is a widespread disease in the human population and mainly causes symptoms affecting the head–neck area and the central nervous system [97]. GPX4 inhibition impaired functions of the cGAS-STING pathway and consequently reduced the anti-HSV ability [98]. It revealed that HSV infection might induce ferroptosis by suppressing the GPX4 system. Moreover, smoking and HPV infection were also reported to inhibit GPX4 and downregulate SLC7A11, respectively [99,100]. Therefore, exposure to both high-risk factor (smoke/alcohol/HPV infection) and HSV infection may lead to excessive ferroptosis and prevent cancer development. Some ncRNAs have been shown to be involved in the modulation of the GPX4 system (see below section) and in HPV infection. It is worth uncovering the mechanism of the ncRNAs between ferroptosis and virus-induced head and neck cancer.

### 3.2. Ferroptosis-Related Non-Coding RNAs in Iron Metabolism

By using miRNA arrays and qPCR, it has been revealed that miR-7-5p was highly expressed in various clinically relevant radioresistant (CRR) cancer cells (established from HeLa, SAS, and hepatocellular carcinoma cells) [50]. Tomita et al. showed that the amount of Fe^2+^ in the mitochondria was markedly downregulated in these cells, suggesting the reduced oxidative stress and ferroptosis in CRR cells [50]. Their work revealed that there were strong relationships between the radioresistance of these cells and the suppression of mitoferrin by miR-7-5p. Mitoferrin-1/2 (Mfrn1 and 2) are members of the mitochondrial solute carrier family of proteins, which are responsible for the transportation of iron into mitochondria [101]. Moreover, their following work demonstrated that knockdown of miR-7-5p led to the downregulation of the iron storage gene (e.g., ferritin) and upregulation of the ferroptosis marker arachidonate lipoxygenase (ALOX) 12 gene, a member of the LOX family [51]. Furthermore, they showed that miR-7-5p regulated the radioresistance via ROS generation, which resulted in ferroptosis.

TfR1 (CD71) and ferritin are two important iron regulators. TfR1, a transmembrane glycoprotein, can internalize the transferrin-bound iron, which is stored in ferritins. Additionally, TfR1 has been validated to serve as a specific ferroptosis marker in tumor cells of multiple cell cultures and tissue [102]. It has been shown that TfR1 is upregulated in liver CSCs and critical to the regulation of erastin-induced cell death of CSCs [103]. In HNSCC, TfR1 has been shown to be widely distributed in proliferating cells [104,105] and serum ferritin can be used to serve as a biomarker of lymph node metastasis [106]. MiR-107 has been found to target TfR1 in colorectal cancer [52] and function as a tumor-suppressor by downregulation of protein kinase C_ε_ in HNSCC [53]. It has been proven that lipid-based nanoparticle delivery of pre-miR-107 reduced the tumorigenicity of HNSCC [54]. Although miR-107 was found downregulated in drug-resistant laryngeal squamous cell carcinoma cells [55] and HNSCC [53], it was reported to be upregulated in NPC and under the modulation of circular RNA transforming growth factor-β receptor II (circTGFBR2) [56]. Another microRNA that regulates TfR1 is miR-148a. It has been revealed that miR-148a can directly bind to TfR1 in hepatocellular carcinoma [57] and repress the progression of OSCC via the ERK/MAPK pathway [58].

LTF is another member of the transferrin family and was found to be a direct binding protein of NEDD4-like E3 ubiquitin-protein ligase (NEDD4L), a novel ferroptosis suppressor [107]. It has been demonstrated that NEDD4L-mediated LTF protein degradation mitigated the iron accumulation and subsequent oxidative damage-associated ferroptotic cell death in multiple types of cancer cells [107]. In addition, LTF was shown to inhibit the cell growth of HNSCC by a p27/cyclin E-dependent pathway [108]. It has also been reported that LTF is downregulated in the NPC tissues and associated with the development of NPC and the radiation resistance effect [109]. NPC patients with low expression of LTF have been indicated to have a poorer prognosis [110]. Furthermore, the upregulated miR-214 in NPC, especially in metastasis-prone NPC tumor tissues, has been revealed to target LTF in NPC specimens [59]. It also has been shown that the targets of miR-214 including LTF, phosphatase and tensin homolog (PTEN), and myocyte enhancer factor 2C (MEF2C) have been verified to participate in the regulation of radiotherapy sensitivity [110].

### 3.3. Ferroptosis-Related Non-Coding RNAs in Lipid Metabolism

Loss of nuclear LKB1 levels has been shown to correlate with HNSCC metastasis [111]. The expression of LKB1 has also been revealed to be downregulated in head and neck cancers, and its promoter region was demethylated or partially methylated [60]. According to the results from the luciferase reporter assay, it has been revealed that miR-100-3p targeted and repressed the mRNA of LKB1 directly in head and neck cancers [60]. Work from Figueroa-González et al. showed that both miR-100-3p and -5p were upregulated in tumor tissues [60] and the role of miR-100-3p in lipid metabolism of cancer is worthy of investigation. ACSL4 is essential to execute ferroptosis by inducing 5-hydroxyeicosatetraenoic acid production [112] and enriching cellular membranes with long polyunsaturated ω6 fatty acids [13]. It has been shown that ACSL4 is a target of miR-106b-5p in brain microvascular endothelial cells [61], and miR-106 has been demonstrated to enhance radiation sensitivity of head and neck cancers [62]. Additionally, ACSL4 was also under the control of miR-424-5p in ovarian cancer [63]. MiR-424-5p is overexpressed in OSCC and promotes epithelial-to-mesenchymal transition and cell migration [64,65].

### 3.4. Ferroptosis-Related Non-Coding RNAs in ROS Accumulation

System xc is composed of light chain, solute carrier family 7 member 11 (SLC7A11/xCT), and heavy chain, solute carrier family 3 member 2 (SLC3A2/CD98). Both subunits help to transport glutamate out of the cell and cystine into the cell and play an important role in ferroptosis. It has been revealed that the expression of SLC3A2 was upregulated in OSCC tissues and related to advanced stages and poor survival of OSCC patients [113]. Moreover, the overexpression of SLC3A2 enhanced migration, invasion, and proliferation of OSCC [113]. It has been shown that lncRNA small nucleolar RNA host gene 1 (SNHG1) promotes the transcription of its neighboring gene SLC3A2 in cis by binding the mediator complex [114] and the expression of SNHG1 was upregulated in NPC [66] and OSCC [67] tissues.

Another subunit SLC7A11 is crucial in the regulation of ferroptosis, redox homeostasis, nutrient dependency, and chemoresistance of various cancers [115,116,117]. It has been shown that the expression of SLC7A11 was upregulated in head and neck cancers [69,100], but was lower in HPV positive head and neck cancers compared to HPV negative tumors [100]. Several microRNAs have been known to modulate SLC7A11 in head and neck cancers. For instance, it has been demonstrated that miR-375 was downregulated in OSCC tissues and it suppressed the expression of SLC7A11 in OSCC cells, which affected cell viability, proliferation, aggressiveness, and induced cell apoptosis and G1/G0 arrest [68]. Another study showed that miR-125b-5p could directly bind to the 3′ UTR of SLC7A11 and inhibit the expression of SLC7A11 in tongue squamous cell carcinoma (TSCC). In addition, Yu et al. demonstrated that the reduced expression of miR-125b-5p was under the regulation of EZH2, and ectopic expression of EZH2 and SLC7A11 mitigated the erastin-induced ferroptosis [69]. Moreover, SLC7A11 also seemed to be modulated by miR-520d-5p in TSCC. It has been revealed that circular RNA circFNDC3B increased the expression of SLC7A11 by acting as a ceRNA of miR-520d-5p and titrating its effect [70]. Yang et al. showed that silencing of circFNDC3B downregulated the expression levels of GPX4 and SLC7A11, and increased ROS, iron, and Fe2+ levels in TSCC cells [70]. Most importantly, they demonstrated that depletion of circFNDC3B retarded the tumor growth in vivo [70].

It has been shown that activating transcription factor 3 (ATF3), a common stress sensor, enhances the erastin-induced ferroptosis by binding to the SLC7A11 promoter and inhibiting SLC7A11 expression in a p53-independent manner [118]. One of the previous studies has revealed that miR-488 suppressed the invasion capacity and the expression of epithelial–mesenchymal transition markers in TSCC by directly targeting ATF3 [71]. Another study showed that circular RNA circ_0001742 upregulated the expression of ATF3 through sequestration of miR-431-5p in TSCC [72]. LncRNA murine retrovirus integration site 1 homolog antisense RNA 1 (MRVI1-AS1) has also been shown to increase the expression of ATF3 by simultaneously inhibiting miR-513a-5p and miR-27b-3p, which enhanced the chemosensitivity of NPC [73]. Likewise, ATF4 has been revealed to confer ferroptosis by binding to the SLC7A11 promoter and activating its expression along with the nuclear factor erythroid 2–related factor 2 (Nrf2), a transcriptional activator of antioxidant genes [119,120]. It has been shown that miR-214 elevated the erastin-induced ferroptosis by decreasing ATF4 expression in hepatoma cells [74], Another microRNA that targets Nrf2 is miR-153, and ferroptosis. It has been shown that the expression levels of both GPX4 mRNA and protein were overexpressed in oral cancer SAS cells, which enhanced cell proliferation [121]. Several non-coding RNAs have been reported to regulate GPX4 in various types of cancers. For instance, miR-15a-3p was found to directly suppress GPX4 in colorectal cancer [79], and miR-15a was upregulated in HPV positive head and neck cancer tissues [80]. Moreover, higher salivary miR-15a-5p can predict a better prognosis for patients with head and neck cancers treated with intensity-modulated radiation therapy [81]. Furthermore, miR-214-3p has been proven to interact and bind to the 3ʹUTR of GPX4 in liver cancer cells [82], and GPX4 is also a direct target of miR-324-3p, which was downregulated in lung adenocarcinoma A549 cells [84]. In NPC tissues, the expression of miR-324-3p was also reduced and ectopic expression of miR-324-3p inhibited migration and invasion of NPC cells [86].

## 4. Conclusions

Although the detailed mechanisms underlying the regulation of ferroptosis by non-coding RNAs remain largely unknown, we are convinced that this review will aid in the development of new investigative tools and therapeutic approaches to attenuate the progression of head and neck cancers.

## Figures and Tables

**Table 1:** Ferroptosis associated-ncRNAs in head and neck cancers.

	Target in Ferroptosis-Related Signaling			Evidence in HNC				
ncRNA	Target	Disease	Reference	Mechanism	Role in HNC	Associated Type of HNC	Model	Reference
Iron Metabolism Associated Target								
miR-7-5p	ROS generation Iron metabolism signaling *ALOX12*	Cervical, liver, and oral cancer	[50,51]	Decrease ROS generation and *ALOX12* Modulate iron metabolism signaling, including increase of *TFRC* and iron storge, and decrease of *SLC25A37* (mitoferrin-1)	Oncogene	Oral SCC	Cell culture	[50,51]
miR-107	*TFRC* *	Colorectal cancer	[52]	Inhibit *PKCɛ* *	Suppressor	Oral SCC	Cell culture Animal model	[53,54]
				Inhibit *HMGB1* *, resulting in the downregulation of autophagy related genes via H19/miR-107/HMGB1 axis	Suppressor	Laryngeal SCC	Cell culture Animal model Clinical specimen	[55]
				Inhibit *TGFBR2* *, resulting in the upregulation of PI3K/AKT signaling and EMT related genes via circTGFBR2/miR-107/ TGFBR2 axis	Oncogene	Nasopharyngeal carcinoma	Cell culture Animal model Clinical specimen	[56]
miR-148a	*TFRC* *	Liver cancer	[57]	Inhibit IGF-IR*, resulting in the downregulation of ERK/MAPK signaling	Suppressor	Oral SCC	Cell culture Clinical specimen	[58]
miR-214	*LTF* *	Nasopharyngeal cancer	[59]	Inhibit LTF*, resulting in the upregulation of AKT activity	Oncogene	Nasopharyngeal carcinoma	Cell culture Animal model Clinical specimen	[59]
**Lipid Metabolism Associated Target**								
miR-100-3p	*LKB1* *	HNC	[60]	Inhibit *LKB1* *^,$^	Oncogene	HNC	Clinical specimen	[60]
miR-106b-5p	*ACSL4* * (H19/miR-106b-5p/ACSL4 axis)	Brain injury after ICH	[61]	Inhibit *RUNX3* * via HPV-E7/DGCR8/miR-106a-5p axis	Suppressor	HPV^+^ or HPV^-^ Hypopharyngeal SCC Oral SCC	Cell culture Clinical specimen	[62]
miR-424-5p	*ACSL4* *	Ovarian cancer	[63]	Inhibit *TGFBR3* *, resulting in the upregulation of NF-κB signaling and EMT related genes	Oncogene	Oral SCC	Cell culture Clinical specimen	[64]
				Inhibit *SOCS2* * via IL-8/STAT5/miR-424-5p axis	Oncogene	Hypopharyngeal SCC Oral SCC	Cell culture Clinical specimen	[65]
**ROS Accumulation Associated Target**								
SNHG1	*SLC3A2*	Colorectal and lung cancer		Increase *NUAK1* *, resulting in the upregulation of Akt activity and EMT related genes via SNHG1/miR-145p-5p/NUAK1 axis	Oncogene	Laryngeal carcinoma Nasopharyngeal carcinoma Oral SCC	Cell culture Clinical specimen	[66,67]
miR-375	*SLC7A11* *	Oral cancer	[68]	Inhibit *SLC7A11* *	Suppressor	Oral SCC	Cell culture Clinical specimen	[68]
miR-125b-5p	*SLC7A11* *	Oral cancer	[69]	Inhibit *SLC7A11* *, resulting in the increase of erastin induced-ferroptosis via EZH2/miR-125b-5p/SLC7A11 axis	Suppressor	Oral SCC	Cell culture	[69]
miR-520d-5p	*SLC7A11* *	Oral cancer	[70]	Inhibit *SLC7A11* *, resulting in the downregulation of GPX4 and promote erastin induced-ferroptosis via circFNDC3B /miR-520d-5p/SLC7A11 axis	Suppressor	Oral SCC	Cell culture Animal model Clinical specimen	[70]
miR-488	*ATF3* *	Oral cancer	[71]	Inhibit *ATF3* *, resulting in the downregulation of EMT via miR-488/ATF3 axis	Suppressor	Oral SCC	Cell culture Clinical specimen	[71]
miR-431-5p	*ATF3* *	Oral cancer	[72]	Inhibit *ATF3* *, resulting in the downregulation of EMT via circ_0001742/miR-431-5p/ATF3 axis	Suppressor	Oral SCC	Cell culture Clinical specimen	[72]
miR-27b-3p miR-513a-5p	*ATF3* *	Breast, lung and nasopharyngeal cancer	[73]	Inhibit *ATF3* *, resulting in the downregulation of Hippo signaling pathway via ATF3/MRVI1-AS1/miR-27b-3p and miR-513a-5p/ATF3 negative feedback loop	Suppressor	Nasopharyngeal carcinoma	Cell culture Animal model Clinical specimen	[73]
miR-214	*ATF4* *	Liver cancer	[74]	Inhibit *Bim* *, resulting in the suppression of cell apoptosis	Oncogene	Nasopharyngeal Carcinoma	Cell culture Animal model Clinical specimen	[75]
miR-93	*Nrf2* *	Breast cancer	[76]	Inhibit *CDKN1A* *, resulting in the increase of cell proliferation	Oncogene	Nasopharyngeal Carcinoma	Cell culture Clinical specimen	[77]
				Inhibit *PD4D4* *	Oncogene	Nasopharyngeal Carcinoma	Cell culture Clinical specimen	[78]
miR-15a-3p	*GPX4* *	Colorectal cancer	[79]	miR-15a is one of the HPV^+^ core miRNAs	-	HNSCC	Clinical specimen	[80]
				miR-15a-5p related to batter prognosis	-	HNSCC	Clinical specimen	[81]
miR-214-3p	*GPX4* *	Liver cancer	[82]	miR-214-3p overexpressed in tumor tissue	-	HNSCC	Clinical specimen	[83]
miR-324-3p	*GPX4* *	Lung cancer	[84]	Inhibit *WNT2B* *, resulting in the suppression of EMT	Suppressor	Nasopharyngeal Carcinoma	Cell culture Clinical specimen	[85,86]

*, represent directed binding by miRNA; ^$^, related to clinical significance; ALOX12, arachidonate lipoxygenase (ALOX) 12 gene; ATF3, activating transcription factor 3; ATF4, activating transcription factor 4; Bim, Bcl-2-interacting mediator of cell death; CDKN1A, cyclin dependent kinase inhibitor 1A; circRNA, circular RNA; DGCR8, DiGeorge syndrome critical region gene 8; EMT, epithelial-mesenchymal transition; GPX4, glutathione peroxidase 4; HMGB1, high mobility group box 1; HNC, head and neck cancer; HNC, head and neck squamous cell carcinoma; HPV, human papillomavirus; ICH, intracerebral hemorrhage; LKB1, liver kinase B1; MRVI1-AS1, murine retrovirus integration site 1 homolog antisense RNA 1; NUAK1, NUAK family kinase 1; PDCD4, programmed cell death 4; PKCɛ, protein kinase Cɛ; ROS, reactive oxygen species; RUNX3, runt-Related Transcription Factor 3; SCC, squamous cell carcinoma; TFRC, transferrin receptor 1; TGFBR2, transforming growth factor-beta receptor type 2; TGFBR3, transforming Growth Factor Beta Receptor 3; WNT2B, Wnt family member 2B.

## Data Availability

Not applicable.
